# Diflunisal in late-onset FAP patients with moderate to severe neuropathy

**DOI:** 10.1186/1750-1172-10-S1-O24

**Published:** 2015-11-02

**Authors:** Laura Obici, Andrea Cortese, Stefano Perlini, Alessandro Lozza, Simona Casarini, Enrico Alfonsi, Giampaolo Merlini

**Affiliations:** 1 Fondazione IRCCS Policlinico San Matteo, Amyloidosis Research and Treatment Center, 27100, Pavia, Italy; 2 Fondazione IRCCS Istituto Neurologico Mondino, General Neurology, 27100, Pavia, Italy; 3 Fondazione IRCCS Policlinico San Matteo, Clinica Medica II, 27100, Pavia, Italy; 4 Fondazione IRCCS Istituto Neurologico Mondino, Neurophysiopathology Unit, 27100, Pavia, Italy

## Background

TTR stabilizers have proved effective in slowing neurological progression in FAP. However, wider experience outside trials is required to further establish their safety profile and clinical benefit in the general FAP population.

## Objective

We evaluated the safety and efficacy of diflunisal (250 mg BID) in late-onset FAP patients with moderate to severe neuropathy and cardiomyopathy treated for at least 24 months.

## Methods

Evaluations included Kumamoto score, polyneuropathy disability score (PND), mBMI, echocardiography and cardiac biomarkers. Adverse events were monitored every three months. Response was evaluated every 12 months.

## Results

24 patients (20 males) affected by FAP associated with 7 different mutations were treated for a median of 24 months (range 12-60). Median age at baseline was 69 years (range 57-82), disease duration 43 months (range 17-90), PND score IIIA (range I-IV), Kumamoto score 25 (2-39), BMI 890 (range 604-1458). 21 patients presented with heart involvement. Median NT-proBNP was 728 pg/ml (range 141-5965), cTnI 0.04 ng/ml (range 0.029-0.65), mLVW 14.2 mm (range 12.5-17.5).

PND increased by 1 point from baseline in 8/18 patients. mBMI remained stable during treatment. Mean change in Kumamoto score was 2.9/year (95% CI -0.3 to 4.8). Cardiac progression occurred only in 2/21 patients. One patient discontinued due to renal failure and three presented with a mild increase in serum creatinine. One patient discontinued after 3 years of treatment due to asymptomatic TnI increase that improved following discontinuation. No GI events were recorded.

## Conclusion

Our results are consistent with the reported beneficial effect of this drug on neurological progression and suggest a favourable impact also on cardiac disease. Potential renal and cardiac toxicity deserves close monitoring.

